# Islet-1 synergizes with Gcn5 to promote MSC differentiation into cardiomyocytes

**DOI:** 10.1038/s41598-020-58387-8

**Published:** 2020-02-04

**Authors:** Hao Xu, Qin Zhou, Qin Yi, Bin Tan, Jie Tian, Xueni Chen, Yue Wang, Xia Yu, Jing Zhu

**Affiliations:** 1 Department of Clinical Laboratory; Ministry of Education Key Laboratory of Child Development and Disorders; National Clinical Research Center for Child Health and Disorders (Chongqing); China International Science and Technology Cooperation base of Child development and Critical Disorders; Children’s Hospital of Chongqing Medical University, Chongqing, P.R. China; 2 Department of Pediatric Research Institute, Ministry of Education Key Laboratory of Child Development and Disorders; National Clinical Research Center for Child Health and Disorders (Chongqing); China International Science and Technology Cooperation base of Child development and Critical Disorders; Children’s Hospital of Chongqing Medical University, Chongqing, P.R. China; 3 Department of Cardiovascular (Internal Medicine), Ministry of Education Key Laboratory of Child Development and Disorders; National Clinical Research Center for Child Health and Disorders (Chongqing); China International Science and Technology Cooperation base of Child development and Critical Disorders; Children’s Hospital of Chongqing Medical University, Chongqing, P.R. China; 4 Chongqing Key Laboratory of Pediatrics, Chongqing, P.R. China; 5 Chengdu Women’s and Children’s Central Hospital, School of Medicine, University of Electronic Science and Technology of China, Chengdu, P.R. China

**Keywords:** Gene expression, Mesenchymal stem cells

## Abstract

Mesenchymal stem cells (MSCs) specifically differentiate into cardiomyocytes as a potential way to reverse myocardial injury diseases, and uncovering this differentiation mechanism is immensely important. We have previously shown that histone acetylation/methylation and DNA methylation are involved in MSC differentiation into cardiomyocytes induced by islet-1. These modifications regulate cardiac-specific genes by interacting with each other in the promoter regions of these genes, but the molecular mechanism of these interactions remains unknown. In this study, we found that the key enzymes that regulate GATA4/Nkx2.5 expression are Gcn5/HDAC1, G9A, and DNMT-1. When α-methylene-γ-butyrolactone 3 (MB-3) was used to inhibit Gcn5 expression, we observed that the interactions among these key enzymes in the GATA4/Nkx2.5 promoters were blocked, and MSCs could not be induced into cardiomyocytes. Our results indicated that islet-1 could induce Gcn5 binding to GATA4/Nkx2.5 promoter regions and induce the interactions among Gcn5, HDAC1, G9A and DNMT-1, which upregulated GATA4/Nkx2.5 expression and promoted MSC differentiation into cardiomyocytes.

## Introduction

Myocardial injury diseases have always been among the highest lethality diseases, primarily due to myocardial cells having no self-renewal ability. Mesenchymal stem cells (MSCs) have been a hot global research topic because of their multiple differentiation potential^1–4^, and they can specifically differentiate into cardiomyocytes^5–7^, which could potentially help cure myocardial injury diseases. Many researchers have successfully induced MSC differentiation into cardiomyocytes through different methods^8–10^, but the molecular mechanism of differentiation is not clear, which results in low induction efficiency and limits the clinical application of MSCs. In our previous research, we overexpressed islet-1 and successfully induced MSC differentiation into cardiomyocyte-like cells that possess cardiac electrophysiological properties. Furthermore, we investigated the molecular mechanism and found that histone modifications and DNA methylation are very important for MSC differentiation; these epigenetic modifications interact with each other during MSC differentiation into cardiomyocytes^11,12^. However, the specific mechanism of the interactions requires further investigation.

Epigenetic modifications exert their function through specific enzymes, and different enzymes modify different sites or have different functions. For example, Gcn5 and CBP/P300 acetylate H3K9/H3K27 sites, Ezh2 methylates H3K27 sites, and Suv39h1 plays a role in H3K9 methylation^13–17^. DNMT-1 is involved in the maintenance of methylation, and DNMT3a/b functions as a de novo methyltransferase^18,19^. However, it remains unclear which specific enzymes are involved in islet-1-induced MSC differentiation into cardiomyocytes and how these enzymes interact with each other. Continuing our previous study, we will further discuss these two issues in this work.

We have confirmed that histone acetylation/methylation and DNA methylation interact with each other in the GATA4 promoter region, coregulate GATA4 expression and induce MSC differentiation into cardiomyocytes^11^. We also found that Gcn5 and DNMT-1 play important roles in regulating GATA4 expression^20^. In this study, we further investigated the specific enzyme that is involved in regulating GATA4 and Nkx2.5 and the molecular mechanism of the epigenetic interaction of these two cardiac-specific transcript factor promoter regions. This research preliminarily proved the epigenetic mechanism by which MSCs differentiate into cardiomyocytes via islet-1 and reveals the key intervention factor for further research. These findings lay the foundation for increasing MSC differentiation rates and improving the clinical application of MSCs.

## Results

### Islet-1 could form a complex with Gcn5 during MSC differentiation into cardiomyocytes

**Figure 1:**
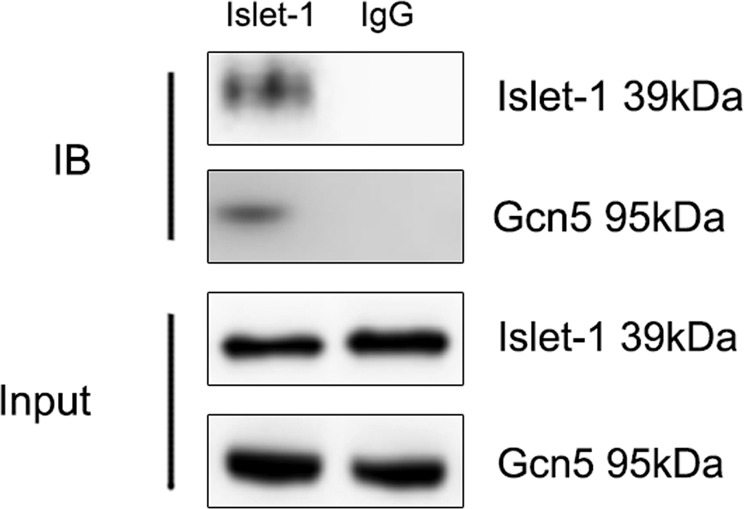
Co-IP experiments confirmed that islet-1 and Gcn5 bound together during MSC differentiation into cardiomyocytes induced by islet-1 overexpression. Islet-1 and IgG antibodies were chosen to pull down proteins, and the islet-1 and Gcn5 bands were then detected by Western blot. Gcn5 was detected in proteins pulled down by the islet-1 antibody, which indicated that islet-1 could form a complex with Gcn5.

We transfected a lentiviral vector containing islet-1 into MSCs, and the transfection efficiency was 81% (Supplementary Figure 1a). Western blot was used to detect islet-1 expression after vector transfection (Supplementary Figure 1b). Next, we used an islet-1 antibody to pull down proteins bound to islet-1 and a Gcn5 antibody to detect the existence of Gcn5 in the pulled down proteins by Western blot. Co-IP results showed that islet-1 and Gcn5 could form a complex after high islet-1 expression was induced in MSCs (Fig. 1). This finding confirms our previous results, which indicated that overexpression of islet-1 could affect histone acetylation to induce MSC differentiation into cardiomyocytes^12^.

### Gcn5 and HDAC1 are key enzymes that regulate histone acetylation levels in the GATA4/Nkx2.5 promoter region

**Figure 2:**
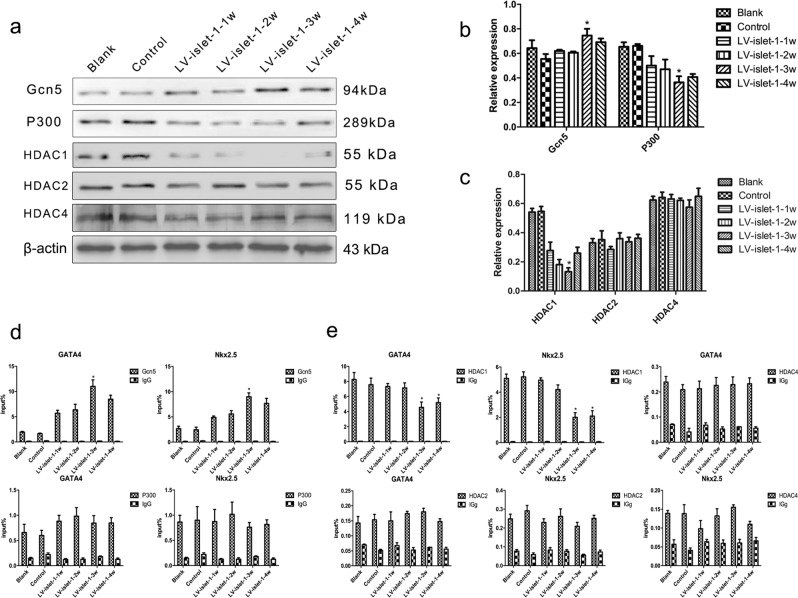
Identification of the key enzymes involved in H3K9 acetylation during MSC differentiation into cardiomyocytes. (**a**) HAT and HDAC bands were detected by Western blot, and the enzymes assessed were Gcn5, P300, HDAC1, HDAC2 and HDAC4. Images were cropped for clarity, and full-length blots/gels are presented in Supplementary Fig. 1. (**b**,**c**) Quantitative analysis of these enzymes. In the process of inducing differentiation, Gcn5 expression slightly increased, P300 and HDAC1 expression decreased, and HDAC2 and HDAC4 expression was not obviously changed. (**d**,**e**) The binding levels of these enzymes in the GATA4/Nkx2.5 promoter were detected by ChIP-qPCR; a normal mouse IgG antibody used as a negative control. The input% showed that the main HAT and HDAC binding in the GATA4/Nkx2.5 promoters was Gcn5 and HDAC1, respectively. *p < 0.05 compared with the blank group. The error bars represent the SD of three independent experiments.

Our previous study showed that H3K9 acetylation in the GATA4/Nkx2.5 promoter region is associated with the expression of the two genes. In this study, we identified the key enzyme that regulates the acetylation level in the GATA4/Nkx2.5 promoter region. Western blot data indicated that the level of Gcn5 was slightly increased, and that of P300 decreased gradually during MSC differentiation into cardiomyocytes induced by islet-1 (Fig. 2a,b). ChIP-qPCR was performed to detect the binding level of Gcn5/P300 in the GATA4/Nkx2.5 promoter region. Additionally, the data showed that the binding level of Gcn5 was increased significantly in both the GATA4 and Nkx2.5 promoter regions, but the binding level of P300 did not change significantly (Fig. 2d). After islet-1 transfection, the trend of Gcn5 binding in the GATA4/Nkx2.5 promoters was consistent with the expression of GAT4/Nkx2.5, which was detected in our previous study^11^. Therefore, we propose that Gcn5 is the key HAT that regulates H3K9 acetylation in the GATA4/Nkx2.5 promoter region.

Because histone acetylation is the result of the balance between HATs and HDACs, HDACs are important for histone acetylation. HDACs restore the positive charge of histones through deacetylation, increasing the attraction between DNA and histones, tightening up the loose nucleosome, and finally suppressing gene expression. Western blot analysis showed that the expression levels of HDAC2 and HDAC4 showed no significant changes, but the expression of HDAC1 gradually decreased after islet-1 transfection (Fig. 2a–c). Correspondingly, the binding level of HDAC1 in the GATA4/Nkx2.5 promoter decreased, as detected by ChIP-qPCR. However, the binding levels of HDAC2/HCAC4 were low in these regions and did not change during MSC differentiation into cardiomyocytes induced by islet-1 (Fig. 2e). These results indicate that the key HDAC that regulates H3K9 acetylation in the GATA4/Nkx2.5 promoter region is HDAC1.

### G9A and DNMT-1 respectively regulated histone methylation and DNA methylation levels in the GATA4/Nkx2.5 promoter region

Histone methylation, as an important histone modification, is involved in inhibiting gene expression and heterochromatin formation. Research has reported that at the H3K9 site, HMTs interact with HATs and affect histone methylation levels in this region^21^. It has been indicated that histone methylation is a vital part of epigenetic modification interactions. G9A and Suv39h1 are the main enzymes involved in H3K9 methylation, and in this study, we measured these two enzymes to confirm their key role in regulating H3K9 methylation in the GATA4/Nkx2.5 promoter region.

**Figure 3:**
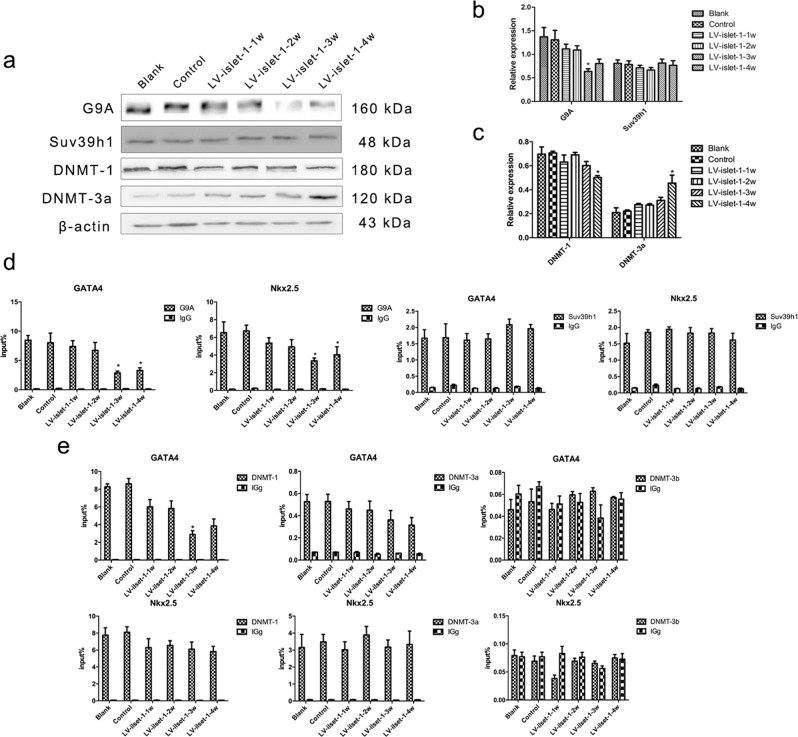
Identification of the key enzymes involved in histone methylation and DNA methylation. (**a**) HMT and DNMT bands were detected by Western blot, and the enzymes assessed were G9A, Suv39h1, DNMT-1, and DNMT-3a. Images were cropped for clarity, and full-length blots/gels are presented in Supplementary Fig. 2. (**b**,**c**) Quantitative analysis of these enzymes. In the process of inducing differentiation, G9A expression decreased gradually, DNMT-3a expression increased, and the other enzymes were not obviously changed. (**d**,**e**) The binding levels of these enzymes in the GATA4/Nkx2.5 promoter were detected by ChIP-qPCR; a normal mouse IgG antibody used as a negative control. The input% showed that the main HMT and DNMT binding in GATA4/Nkx2.5 promoters was G9A and DNMT-1, respectively. *p < 0.05 compared with the blank group. The error bars represent the SD of three independent experiments.

Western blot analysis was used to detect G9A/Suv39h1 expression during MSC differentiation into cardiomyocytes induced by islet-1. The results showed that the expression of G9A decreased gradually and was lowest in the LV-islet-1-3w group, but the expression of Suv39h1 did not change after islet-1 transfection compared with that in the blank and control groups (Fig. 3a,b). ChIP-qPCR indicated that in the GATA4/Nkx2.5 promoter region, the binding level of G9A was higher than that of Suv39h1; furthermore, after islet-1 transfection, the binding level of G9A in both the GATA4 and Nkx2.5 promoters decreased gradually and was lowest in the LV-islet-1-3w group, which was consistent with the H3K9 methylation level in the GATA4/Nkx2.5 promoter regions detected in our previous study. However, the binding level of Suv39h1 did not obviously change during the period of differentiation (Fig. 3d). These results suggest that G9A is the key HMT involved in regulating the histone methylation level in the GATA4/Nkx2.5 promoter region during MSC differentiation into cardiomyocytes.

DNA methylation is best known for its role in gene silencing. It can alter gene expression without changing a gene’s base sequence. We used Western blot to detect the expression of DNMTs, and the results showed that the level of DNMT-1 decreased slightly and that the amount of DNMT-3a increased at the 4th week after islet-1 transfection; however, the expression of DNMT-3b was too low to detect (Fig. 3a–c). Furthermore, our ChIP-qPCR results indicated that the binding level of DNMT-1 in the GATA4/Nkx2.5 promoters was much higher than that of DNMT-3a, and the binding level of DNMT-3b was as low as that of the negative control. In addition, the binding level of DNMT-1 decreased gradually in the GATA4 promoter region during MSC differentiation into cardiomyocytes but did not change in the Nkx2.5 promoter region (Fig. 3e). These results suggest that DNMT-1 is the key DNMT responsible for regulating the DNA methylation level in the GATA4 promoter. Additionally, the binding level of DNMT-1 did not change in the Nkx2.5 promoter region, which explains why the methylation level of the Nkx2.5 promoter was not affected by transfection with islet-1, which was found in our previous study^11^.

### HP1 associated with the DNA methylation level in the GATA4/Nkx2.5 promoter region

**Figure 4:**
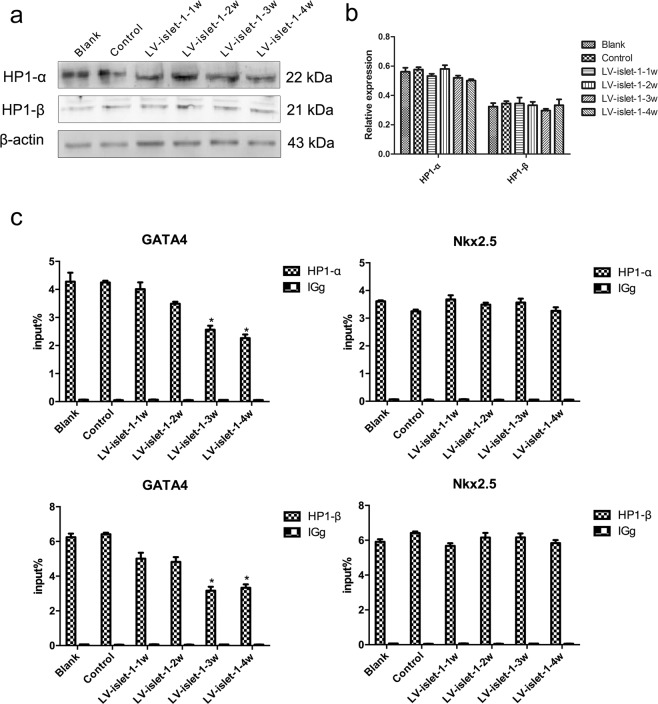
The expression of HP1 and its binding level in the GATA4/Nkx2.5 promoters. (**a**) HP1α and HP1β bands detected by Western blot. Images were cropped for clarity, and full-length blots/gels are presented in Supplementary Fig. 3. (**b**) Quantitative analysis of HP1α and HP1β. In the process of inducing differentiation, HP1α and HP1β expression was not obviously changed. (**c**) ChIP-qPCR detection of the binding level of HP1α and HP1β in GATA4/Nkx2.5 promoters. A normal mouse IgG antibody was used as a negative control. The input% showed that both HP1-α and HP1-β could bind to GATA4/Nkx2.5 promoters, but their binding level changed in only the GATA4 promoter. *p < 0.05 compared with the blank group. The error bars represent the SD of three independent experiments.

HP1 is a component of heterochromatin that recognizes and binds the methylated H3K9 site, leading to epigenetic repression. DNMT-1 could be recruited to the methylated H3K9 site by HP1 and perform DNA methylation. Therefore, HP1 plays an important role in the interaction of histone methylation and DNA methylation. We found that the expression levels of HP1α and HP1β were not changed during MSC differentiation into cardiomyocytes (Fig. 4a,b). However, our ChIP-qPCR results showed that the binding levels of HP1α and HP1β decreased in the GATA4 promoter after islet-1 transfection but did not change in the Nkx2.5 promoter (Fig. 4c). The ChIP-qPCR results were consistent with the binding levels of DNMTs. We think the reason that the DNA methylation level in the Nkx2.5 promoter was unchanged is that HP1 binding in the Nkx2.5 promoter is stable, which recruits DNMT-1 to this region and maintains DNA methylation.

### Gcn5 inhibition does not affect the expression of other key enzymes but affects their binding level in the GATA4/Nkx2.5 promoter

**Figure 5:**
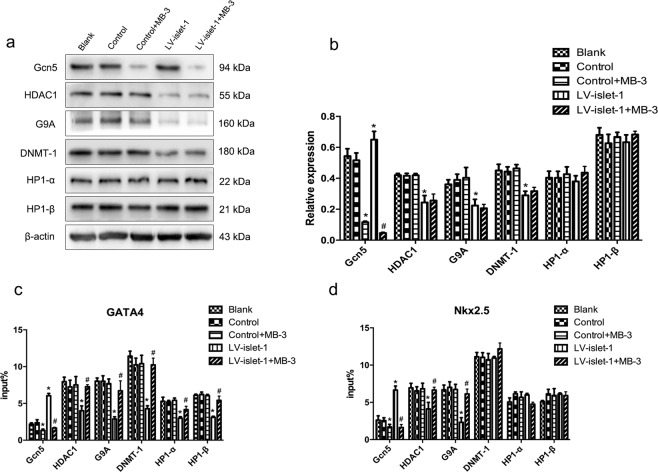
During MSC differentiation into cardiomyocytes induced by Islet-1, the effects of Gcn5 inhibition on other key enzymes were assessed. (**a**) Western blot was used to detect the expression of the enzymes involved in regulating GATA4/Nkx2.5 after Gcn5 was inhibited by MB-3. The enzymes assessed were Gcn5, HDAC1, G9A, DNMT-1 and HP1. Images were cropped for clarity, and full-length blots/gels are presented in Supplementary Fig. 4. (**b**) Quantitative analysis of these enzymes. MB-3 inhibited Gcn5 expression in both the Control + MB-3 and LV-islet-1 + MB-3 groups but had no effect on the other enzymes. (**c**,**d**) ChIP-qPCR results of the binding levels of these enzymes in the GATA4/Nkx2.5 promoters after Gcn5 was inhibited by MB-3 during MSC differentiation. After islet-1 transfection and Gcn5 inhibition, the binding level of the other enzymes in the GATA4/Nkx2.5 promoters did not change compared with those in the blank, control and control + MB-3 promoters. *p < 0.05 compared with the blank group, #p < 0.05 compared with the LV-islet-1 group. The error bars represent the SD of three independent experiments.

Of all the key enzymes that regulate GATA4/Nkx2.5 expression, Gcn5 is the only enzyme with upregulated expression and an increased binding level during MSC differentiation induced by islet-1. To confirm the interaction among the three epigenetic modifications, we inhibited Gcn5 expression and detected changes in the other key enzymes. We used the Gcn5-specific inhibitor butyrolactone 3 (MB-3) to suppress Gcn5 expression after islet-1 transfection. The Western blot results showed that the expression of Gcn5 was significantly suppressed, but the expression of other key enzymes, HDAC1, G9A DNMT-1 and HP1α/β, was not changed in the LV-islet-1 + MB-3 group (Fig. 5a,b). This result indicates that the expression levels of these key enzymes were not affected by Gcn5 inhibition. ChIP-qPCR detected the binding levels of key enzymes in the GATA4/Nkx2.5 promoters after Gcn5 inhibition. The results showed that compared with that of the LV-islet-1 group, the binding level of Gcn5 was significantly decreased due to MB-3 inhibiting Gcn5 expression; as the response of Gcn5 was blocked, the binding of the other enzymes was all increased in the GATA4 promoter region (Fig. 5c). In comparison, the binding levels of DNMT-1 and HP1α/β were not affected by Gcn5 inhibition in the Nkx2.5 promoter region (Fig. 5d). These data suggest that Gcn5 interacts with these other enzymes in the GATA4 promoter region; furthermore, the fact that DNMT-1 and HP1α/β binding to the Nkx2.5 promoter was not affected by Gcn5 inhibition suggests that the interaction of these key enzymes is different in the GATA4 and Nkx2.5 promoters.

### Histone acetylation/methylation and DNA methylation in the GATA4 promoter changed after Gcn5 inhibition

**Figure 6:**
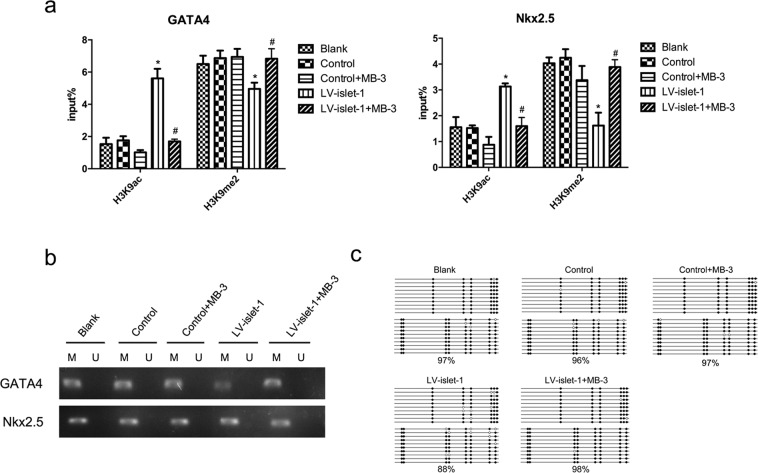
During MSC differentiation into cardiomyocytes induced by islet-1, changes in the levels of H3K9 acetylation/methylation and DNA methylation in the GATA4 and Nkx2.5 promoters after Gcn5 inhibition were detected. (**a)** ChIP-qPCR was used to detect H3K9 acetylation and methylation levels in the GATA4 and Nkx2.5 promoters. The input% showed H3K9 acetylation and methylation levels in the LV-islet-1 + MB-3 group, in contrast to those in the LV-islet-1 group. *p < 0.05 compared with the blank group, ^#^p < 0.05 compared with the LV-islet-1 group. The error bars represent the SD of three independent experiments. (**b**) MSP was used to detect the DNA methylation level in the GATA4 and Nkx2.5 promoters. Two pairs of primers were designed by Methprimer: one set of methylation-specific primers and one set of nonmethylation-specific primers. Images were cropped for clarity, and full-length blots/gels are presented in Supplementary Fig. 5. (**c**) Bisulfite sequencing analysis of the GATA4 promoter. Methylated CG (filled circles) and unmethylated CG (open circles) are indicated. The methylation rate of the GATA4 promoter was 88% in the LV-islet-1 group and 98% in the LV-islet-1 + MB-3 group, which were the same as those in the blank and control groups.

We investigated the effect of Gcn5 inhibition on histone acetylation/methylation and the DNA methylation level in the GATA4/Nkx2.5 promoters. The ChIP-qPCR data showed that compared with those in the LV-islet-1 group, the histone acetylation levels of the GATA4 and Nkx2.5 promoters were decreased after Gcn5 inhibition, but the histone methylation levels were increased to the levels of the blank and control groups (Fig. 6a). On the other hand, we detected the DNA methylation level of the GATA4/Nkx2.5 promoter through MSP. The results indicate that the DNA methylation level of the Nkx2.5 promoter was not affected by Gcn5 inhibition, but in the GATA4 promoter, this level was increased compared with that in the LV-islet-1 group (Fig. 6b). The BSP results showed that the DNA methylation level of the GATA4 promoter increased to 98% from 88% after Gcn5 inhibition (Fig. 6c). These results suggest that in the GATA4 promoter region, the inhibition of Gcn5 affected not only the histone acetylation level but also the histone methylation and DNA methylation levels. This is consistent with the binding level of key enzymes in the GATA4 promoter after Gcn5 inhibition. Similarly, the DNA methylation level in the Nkx2.5 promoter did not change after Gcn5 inhibition because Gcn5 inhibition did not affect the binding of HP1α/β and DNMT-1 to the Nkx2.5 promoter.

### Islet-1 could not induce MSC differentiation into cardiomyocytes after Gcn5 inhibition

**Figure 7:**
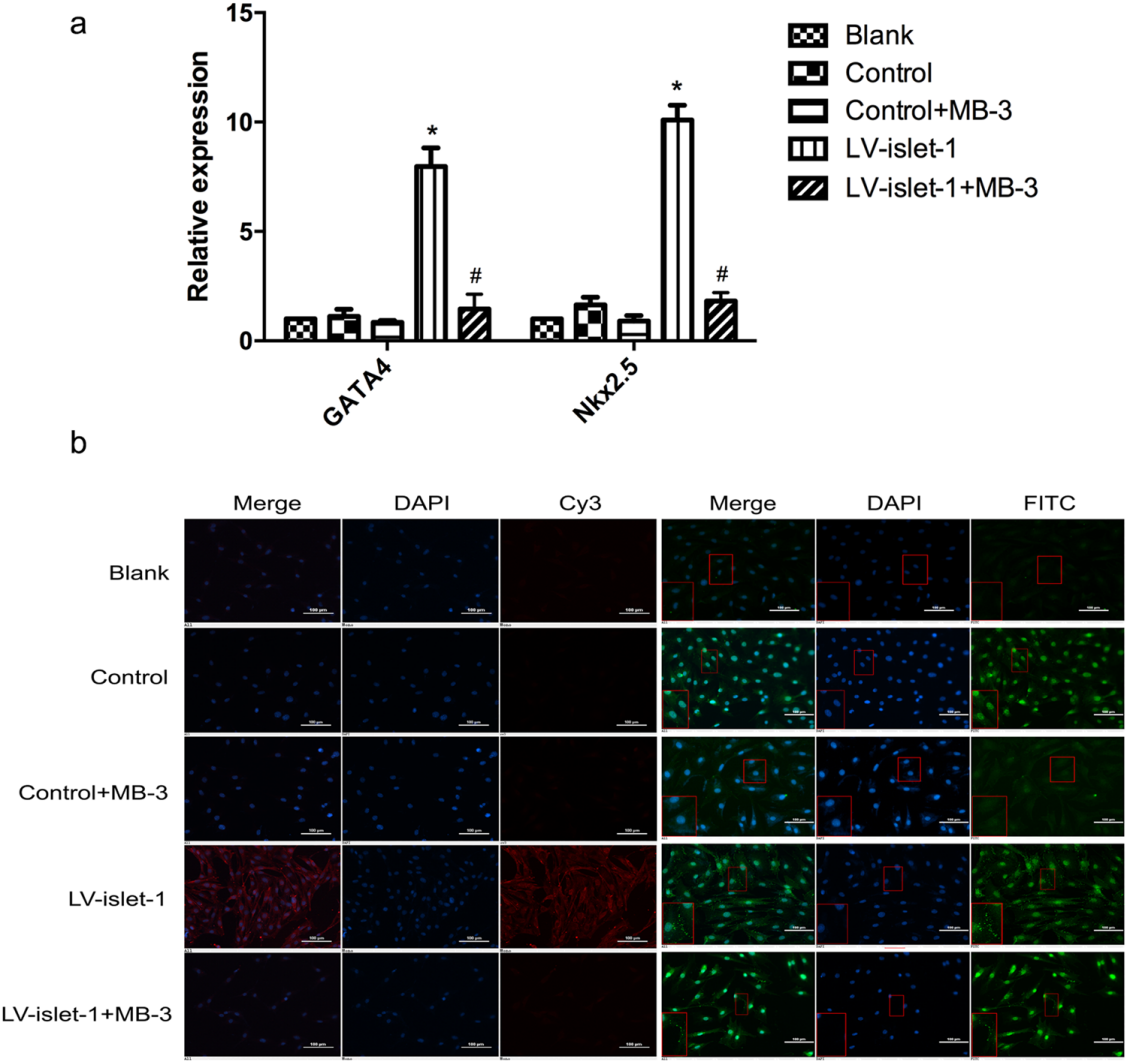
The effect of Gcn5 inhibition on MSC differentiation into cardiomyocytes induced by islet-1. (**a**) FQ-PCR detected the expression levels of GATA4 and Nkx2.5 after Gcn5 was inhibited. GATA4 and Nkx2.5 expression increased after islet-1 transfection, but in the LV-islet-1 + MB-3 group, their expression did not change compared with that in the blank and control groups. *p < 0.05 compared with the blank group, ^#^p < 0.05 compared with the LV-islet-1 group. The error bars represent the SD of three independent experiments. (**b**) An immunofluorescence assay was used to detect the expression of cTnT and Cx43 (100×). The fluorescent dye of cTnT was CY3, that of Cx43 was FITC, and DAPI was used for nuclear staining. cTnT express in Cytoplasm, and only expressed in LV-islet-1 group; Cx43 express mainly in Membrane, and expressed much more in LV-islet-1 group than other groups. The block in the lower left corner of a single image is the local amplification (200×) of the corresponding positions.

In our previous study, islet-1 overexpression promoted MSC differentiation into cardiomyocytes by increasing cardiac-specific gene (GATA4/Nkx2.5) expression. In this study, compared with that in the blank and control groups, the expression of GATA4/Nkx2.5 was not changed after Gcn5 inhibition, even when islet-1 was overexpressed (Fig. 7a). We thought islet-1 could induce MSC differentiation into cardiomyocytes because MSCs began to express the cardiac-specific gene cTnT after islet-1 transfection, and the myocardium electrophysiological properties of MSCs may be related to Cx43 expression. However, through immunofluorescence, we found that after Gcn5 inhibition, islet-1 could not induce MSC expression of cTnT and Cx43 (Fig. 7b). These results suggest that MSCs could not be induced into cardiomyocytes after Gcn5 inhibition, even in the presence of islet-1.

## Discussion

Epigenetic manipulation to differentiate MSCs into specialized cells has the potential to serve regenerative purposes. Various studies support the involvement of epigenetic mechanisms through gene expression control, stem cell self-renewal and lineage fate determination^22,23^. During differentiation into a particular lineage, the specific genes of this lineage undergo active transcription, and the genes responsible for self-renewal and pluripotency are repressed^22,24^. This on-off mechanism is associated with posttranslational modifications, especially histone acetylation/methylation and promoter DNA methylation. These epigenetic modifications are critical for regulating gene expression, and many studies have focused on one of them. However, in practice, regulating gene expression is extensively coordinated by a network of many factors working together^21,25–27^, which is far beyond the capability of a single epigenetic modification. In the complicated differentiation process, regulating gene expression may be more involved. Different epigenetic modifications probably regulate the same gene through interactions with each other^28–30^. Regarding MSC differentiation, it will be helpful to investigate the mechanism of interactions between epigenetic modifications.

Our previous study reported that histone acetylation/methylation and DNA methylation are involved in MSC differentiation into cardiomyocytes by regulating GATA4 and Nkx2.5 expression, and these modifications showed obvious interactions with each other in the GATA4/Nkx2.5 promoter. DNA methylation was not involved in regulating Nkx2.5 expression and did not interact with the other two modifications in the Nkx2.5 promoter^11^. The interactions among histone acetylation, histone methylation and DNA methylation work in different ways in the GATA4 and Nkx2.5 promoters, but the details are not completely understood. GATA4 and Nkx2.5 are critical transcription factors in heart development, and elucidating the molecular mechanism will contribute to regulating their expression and help in the development of a method to differentiate MSCs into cardiomyocytes.

In this study, we found that the key enzymes of H3K9 acetylation, H3K9 methylation and DNA methylation in the GATA4/Nkx2.5 promoters are Gcn5/HDAC1, G9A and DNMT-1, respectively. During the process of MSC differentiation induced by islet-1, the binding level of Gcn5 in the promoter region of GATA4/Nkx2.5 was obviously increased. On the other hand, the binding levels of G9A, DNMT-1, and HDAC1 were all decreased in the GATA4 promoter, but that of DNMT-1 in the Nkx2.5 promoter was not changed. When the cells were treated with the Gcn5 inhibitor MB-3, the expression levels of other key enzymes (HDAC1, G9A, and DNMT-1) were unaffected, and their binding levels in the GATA4/Nkx2.5 promoters were also unchanged. This indicated that the changes in these key enzymes were not caused by islet-1 but by Gcn5 interacting with them. There is another critical factor involved in the interactions among these key enzymes, HP1^31–33^. HP1 can bind to the methylated H3K9 site and recruit DNMT-1 binding to this region; thus, the CpG sites of this region are methylated^34–36^. When MSCs differentiated into cardiomyocytes, the H3K9 methylation level in the GATA4 promoter was decreased, and the binding of HP1 to this region was reduced as well. However, HP1 binding to the Nkx2.5 promoter was unaffected, and even the H3K9 methylation level of this region was decreased. This could be the reason why DNMT-1 binding to the Nkx2.5 promoter was unaffected during MSC differentiation.

**Figure 8:**
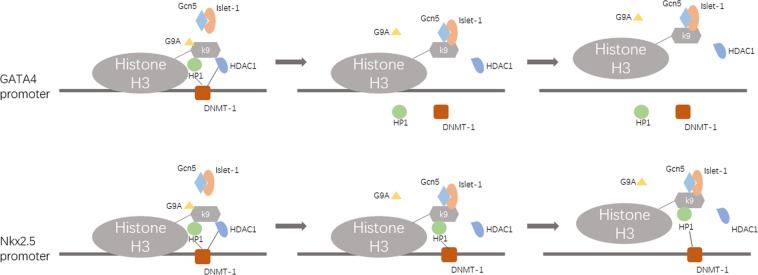
The epigenetic modification model of MSC differentiation into cardiomyocytes induced by islet-1. Islet-1 guides Gcn5 binding to the GATA4 and Nkx2.5 promoters, and Gcn5 interacts with G9A, HP1, DNMT-1 and HDAC1. Then, the chromatin of the GATA4 and Nkx2.5 promoter region loosened because of the hyperacetylation of these regions, which is conducive to GATA4 and Nkx2.5 expression.

In our study, when islet-1 was used to induce MSC differentiation into cardiomyocytes, Gcn5 expression was slightly increased, and its binding level in the GATA4 promoter was significantly increased. On the other hand, research has reported that Gcn5 and islet-1 could form a complex^37^. Therefore, we thought that islet-1 could increase GATA4/Nkx2.5 expression by combining with Gcn5 and guiding it to the GATA4/Nkx2.5 promoters instead of by increasing Gcn5 expression. Based on the above results, we propose an epigenetic modification model of MSC differentiation into cardiomyocytes induced by islet-1 (Fig. 8). First, the overexpressed islet-1 could guide more Gcn5 to bind to the GATA4 promoter; then, the H3K9 site of this region may be hyperacetylated. After that, as more Gcn5 binds to the GATA4 promoter, more G9A is replaced, and the H3K9 site becomes hypomethylated. Then, the binding level of HP1 to the GATA4 promoter decreases because of H3K9 hypomethylation in this region, which simultaneously frees DNMT-1 from the GATA4 promoter, and the CpG methylation level of this region decreases. Furthermore, HDAC1 is replaced by Gcn5 from the H3K9 site of the GATA4 promoter. However, according to the literature, DNMT-1 can recruit HDAC1. Therefore, HDAC1 binding to the GATA4 promoter further decreases because DNMT-1 is lost from this region. This phenomenon further increases the H3K9 acetylation level of the GATA4 promoter. Finally, the chromatin structure of the GATA4 promoter region becomes loose, which is conducive to GATA4 expression. The interactions among these key enzymes in the Nkx2.5 promoter is different from those in the GATA4 promoter. HP1 binding to the Nkx2.5 promoter is unaffected by the H3K9 methylation level of this region, which ensures that DNMT-1 binds to this region stably. In the process of induced differentiation, we found significant differences in some parameters, such as the binding levels of Gcn5 and DNMT-1 in the GATA4/Nkx2.5 promoters, which were lost at the 4th week; at this time point, we also found that the protein expression of islet-1 decreased (Supplementary Figure 2),we were not sure whether these changes were associated with the reduction of Islet-1. We thought that it may be a trend of differentiation progress, and the specific meanings of these changes require our further study.

Our results showed that when Gcn5 was inhibited, the interactions among these key enzymes were blocked, the expression of GATA4 and Nkx2.5 was repressed, and islet-1 could not induce MSC differentiation into cardiomyocytes. These results indicated that the epigenetic modifications and the interactions among these key enzymes are critical for MSC differentiation. However, in practice, the mechanism of MSC differentiation is more complicated. For example, in addition to epigenetic modifications, the stemness of MSCs and the energy metabolism conversion during MSC differentiation are equally important. By studying these two topics, we can thoroughly understand the mechanism of MSC differentiation.

## Methods

### Cell culture, lentiviral vector transfection and MB-3 treatment

C3H10T1/2 cells (University of Chicago Molecular Oncology Laboratory, Chicago, IL, USA) were cultured in DMEM supplemented with 10% foetal bovine serum (FBS; Millipore, USA), lentiviral vectors containing islet-1 or GFP (MOI = 20, GENECHEM, Shanghai, China) and 5 µg/ml polybrene. The culture medium was replaced after 24 h of incubation at 37 °C in 5% CO2. The cells were divided into three groups: a blank group (C3H10T1/2), a control group (lentiviral vector with GFP), and an LV-islet-1 group (lentiviral vector with islet-1). According to the transfection duration, the LV-islet-1 group was further divided into four subgroups: LV-islet-1-1w, LV-islet-1-2w, LV-islet-1-3w, and LV-islet-1-4w. Butyrolactone 3 (MB-3, 100 µmol, Santa Cruz, sc-358657) was added to the culture medium after LV-islet-1 or GFP transfection for 24 h; the treatment was applied for 4 weeks, and the cells were defined as the LV-islet-1 + MB-3 group and the control + MB-3 group. The culture medium of all groups was changed every 2–3 days.

### Co-immunoprecipitation (Co-IP) assay

Co-immunoprecipitation was conducted using a Co-IP assay kit (Merck Millipore, DA, Germany), and the total protein concentrations of the cell lysates were measured with a BCA assay. An anti-islet-1 (Abcam, Cambridge, MA, UK) antibody was used to pull down islet-1, and an anti-IgG antibody was used as a negative control. Protein collection was analysed by Western blot using an anti-Gcn5 (Epigentek, Farmingdale, NY, USA) antibody to detect its existence in islet-1-recruiting proteins.

### Chromatin immunoprecipitation (ChIP)-qPCR assay

Cells were resuspended in PBS; formaldehyde (1%) was added to the samples to crosslink protein-DNA complexes for 5 min at room temperature, and glycine was then added to a final concentration of 0.125 M for 5 min to terminate the crosslinking. The crosslinked material was fragmented by sonication (UCD-200, Bioruptor), which consisted of 25 cycles of 30 s each time with 30-s cooldown intervals. ChIP was performed using a ChIP assay kit (Merck Millipore, DA, GER), and the antibodies used are listed in Supplementary Table 1. The experiment had both a positive control group (precipitated with an anti-RNA polymerase II antibody) and a negative control group (precipitated with normal mouse IgG); 1% of the starting chromatin was used as an input. The primers were designed as follows: Nkx2.5 (forward) 5′-ACCGCCTGGGTGATAGAC-3′, Nkx2.5 (reverse) 5′-CCCTCCCGAGATTGAAGAT-3′, GATA4 (forward) 5′-GCTACAGGGAGTGATGAGAAG-3′, and GATA4 (reverse) 5′-CACCAGCCCAGGAGTTTAT-3′. The qPCR reaction settings were as follows: step 1, 95 °C for 5 min; step 2, 40 cycles of 95 °C for 15 s and 60 °C for 30 s; step 3, 65 °C for 5 s and 95 °C for 0.5 s. To calculate the input%, the following equation was used: Ct(input)−6.644; then, the input% was adjusted to 100% as follows: 100*2^ (Adjusted Ct (input) - Ct (IP)).

### Protein extraction and western blot

Proteins were extracted from cells using RIPA Reagent (P0013B, Beyotime Biotech, China). Protein samples were mixed with 5× buffer and boiled for 5 min before being loaded onto a 10% SDS-PAGE gel. After electrophoresis, the proteins were transferred to polyvinylidene fluoride (PVDF) membranes (Millipore, USA). According to the markers, the membranes were cut into pieces and incubated in 5% nonfat milk-PBST for 1 h. The membranes were incubated with primary antibodies (Supplementary Table 1) overnight at 4 °C and then washed with PBST 3 times for 10 min. After that, the membranes were incubated with the corresponding secondary antibody. Positive bands were detected by chemiluminescent reactions (Millipore, USA).

### Bisulfite sequencing PCR (BSP) assay

DNA was extracted from cells using a DNA extraction kit (AP-MN-MS-GDNA-250, Axygen) and then treated with bisulfite (ZYMO RESEARCHA, California, USA). The PCR products were extracted from the gel (AP-GX-250, Axygen) and ligated into the pMD®18-T Vector (D101B, TAKARA). Plasmid-transformed DH5α bacteria were cultured overnight, and plasmid DNA was isolated (Axygen). At least 10 separate clones were chosen for sequence analysis.

### Genomic DNA extraction and methylation-specific PCR (MSP)

Genomic DNA was collected using a genomic DNA purification kit (TIANGEN, Beijing, China) and modified using an EZ DNA Methylation-Gold Kit (ZYMO RESEARCHA, California, USA). The primers for methylation-specific PCR (MS-PCR) were designed as follows: methylation-specific primers, Nkx2.5 (forward) 5′-ATTAGGTGACGTAGAATTGTTCGTC-3′, Nkx2.5 (reverse) 5′-CGCCTCTCTACCCTAAATATAACG-3′; GATA4 (forward) 5′-GGGTTTATAGGTATTGACGTCGA-3′, and GATA4 (reverse) 5′-GATAAAAACTACAAAACGCCGAA-3′. The nonmethylation-specific primers were Nkx2.5 (forward) primer 5′-TAGGTGATGTAGAATTGTTTGTTGT-3′, Nkx2.5 (reverse) primer 5′-CCACCTCTCTACCCTAAATATAACAC-3′; GATA4 (forward) 5′-AGGGTTTATAGGTATTGATGTTGA-3′, and GATA4 (reverse) 5′-CCAATAAAAACTACAAAACACCAAA-3′. The PCR products were run on a 2% agarose gel, stained with ethidium bromide, and evaluated under UV light.

### Total RNA extraction and real-time PCR

The total RNA samples were collected using an RNA extraction kit (RP120; BioTeke) and transformed into cDNA using a PrimeScript RT reagent kit (Takara, Dalian, Liaoning, China). Then, the resulting cDNA samples were amplified with gene-specific primers and a SYBR Green dye kit (Takara, Dalian, Liaoning, China). Primers were designed as follows: Nkx2.5 (forward) 5′-GAGCCTGGTAGGGAAAGAGC-3′, Nkx2.5 (reverse) 5′-GGTGGGTGTGAAATCTGAGG-3′, GATA4 (forward) 5′-GACTACCACCACCACGCTGT-3′, and GATA4 (reverse) 5′-ATTCAGGTTCTTGGGCTTCC-3′.

### Immunofluorescence

The cells were fixed in 4 °C acetone for 15 min and blocked with goat serum (1:20). Then, primary anti-cardiac troponin T monoclonal (1:400, Abcam) and anti-connexin 43 polyclonal (1:100, Abcam) antibodies were added for overnight incubation at 4 °C. Next, secondary antibodies (1:150; CoWin Bioscience, Beijing, China) conjugated to Cy3 were added and incubated for 1 h at 37 °C. DAPI was then added for 3 min. Images were acquired under a fluorescence microscope (BX51; Olympus).

### Statistical analysis

Each experiment was repeated at least three times. All data are expressed as the means ± SD, while statistical evaluations were performed using independent samples and t-tests, continuity correction chi-square tests and one-way ANOVA. SPSS 17.0 software (SPSS Inc., Armonk, NY, USA) was used for statistical analysis. A value of P < 0.05 was considered statistically significant for all analyses.

## Supplementary information

supplementary information.

## Data Availability

All data used during the current study available from the corresponding author on reasonable request.
