# Supplementary material for: Islet-1 synergizes with Gcn5 to promote MSC differentiation into cardiomyocytes
Source: Sci Rep. 2020 Feb 4;10:1817. doi: 10.1038/s41598-020-58387-8 (PMC7000709; doi:10.1038/s41598-020-58387-8)
Supplement: Supplementary file 1 — supplementary information. [file 41598_2020_58387_MOESM1_ESM.pdf]

Islet-1 synergizes with Gcn5 to promote the differentiation of MSCs into cardiomyocytes

Hao Xu, Qin Zhou, Bin Tan, Qin Yi, Jie Tian, Xueni Chen, Yue Wang, Xia Yu, Jing Zhu

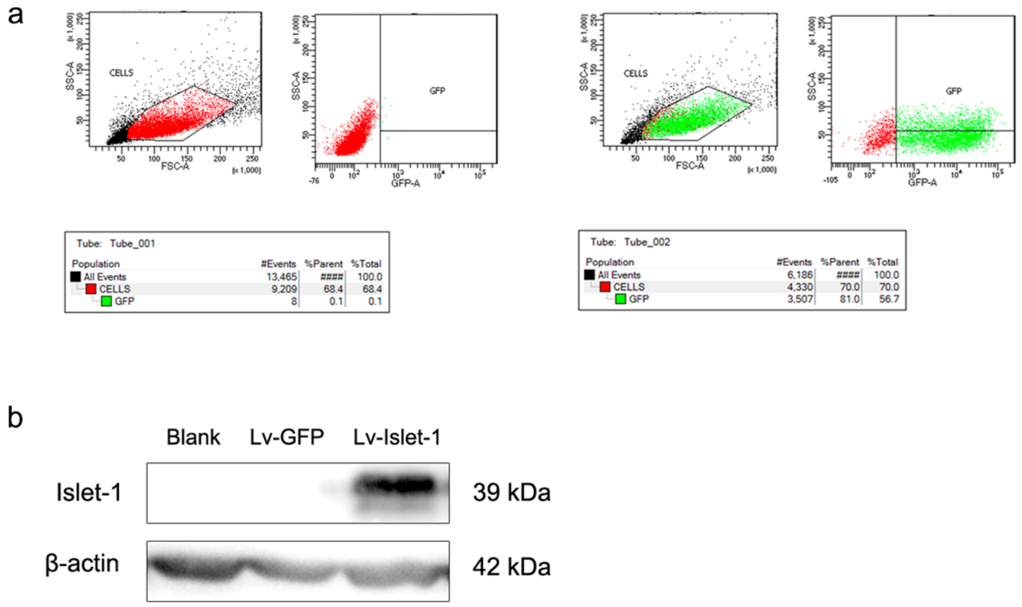

Supplementary Figure 1 The transfection efficiency of Lentiviral vectors detected by Flow Cytometry; Western Blot detect the expression of islet-1 after transfection. (a) Transfection efficiency (81%) was detected by flow cytometry. (b) The protein expression of Islet-1 was detected by Western blot; Islet-1 was highly expressed in the experimental group.

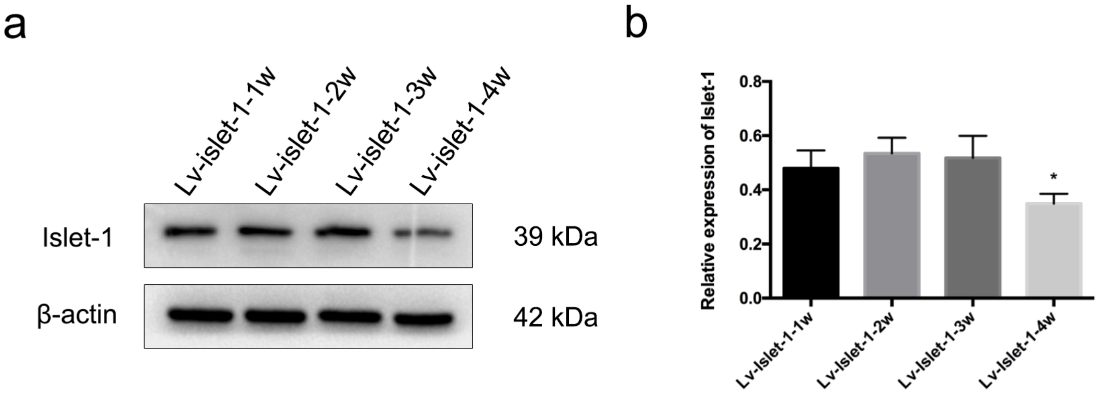

Supplementary Figure 2 a. The protein expression of Islet-1 was detected by Western blot after transfection with Lentiviral vectors. b. After 4 weeks, islet-1 remains over-expressed, but decreased slightly compared with first three weeks, and there were no differences between first three weeks. *$p < 0.05$ compared with Lv-islet-1-1w, -2w and -3w group. The error bars represent the SD of three independent experiments.

a

b

c

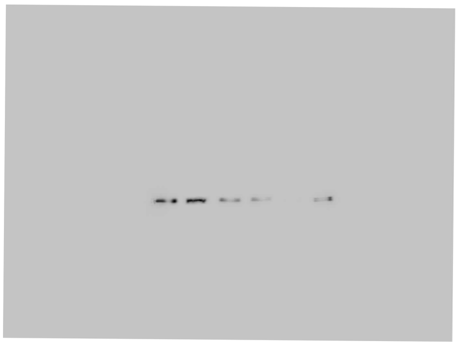

d

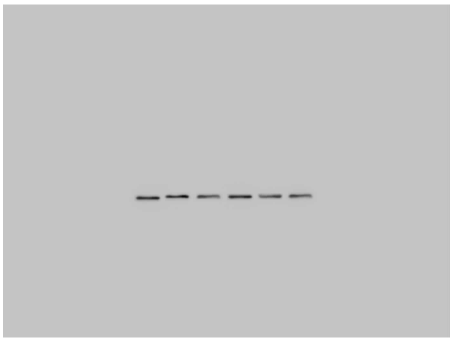

e

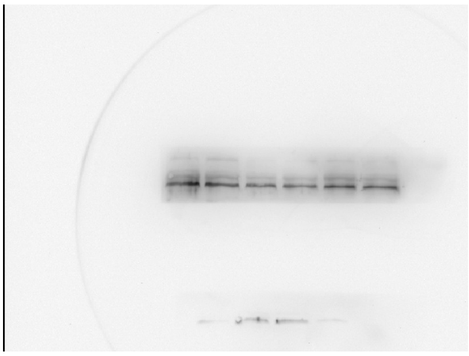

Supplementary Figure 3 Full-length blots/gels for Figure 2. (a) Corresponding to Figure 2a: Gcn5. (b) Corresponding to Figure 2a: P300. (c) Corresponding to Figure 2a: HDAC-1. (d) Corresponding to Figure 2a: HDAC-2. (e) Corresponding to Figure 2a: HDAC-4.

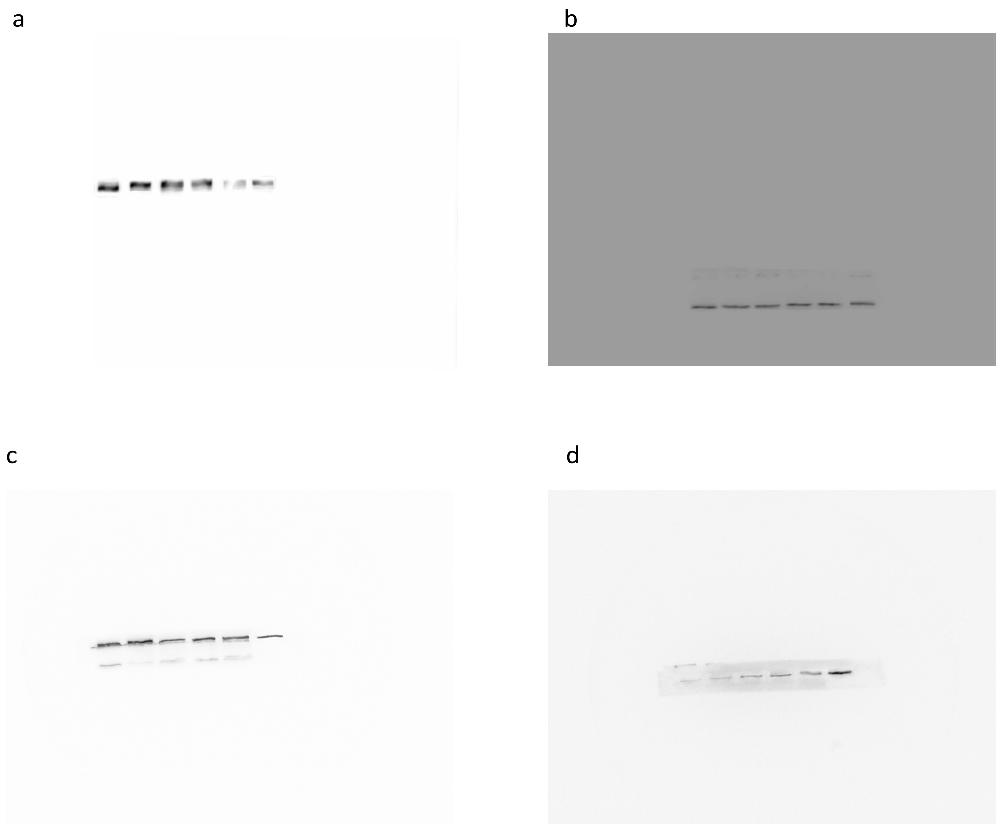

Supplementary Figure 4 Full-length blots/gels for Figure 3. (a) Corresponding to Figure 3a: G9A. (b) Corresponding to Figure 3a: Suv39h1. (c) Corresponding to Figure 3a: DNMT-1. (d) Corresponding to Figure 3a: HDAC-2. (d) Corresponding to Figure 3a: DNMT-3a.

a

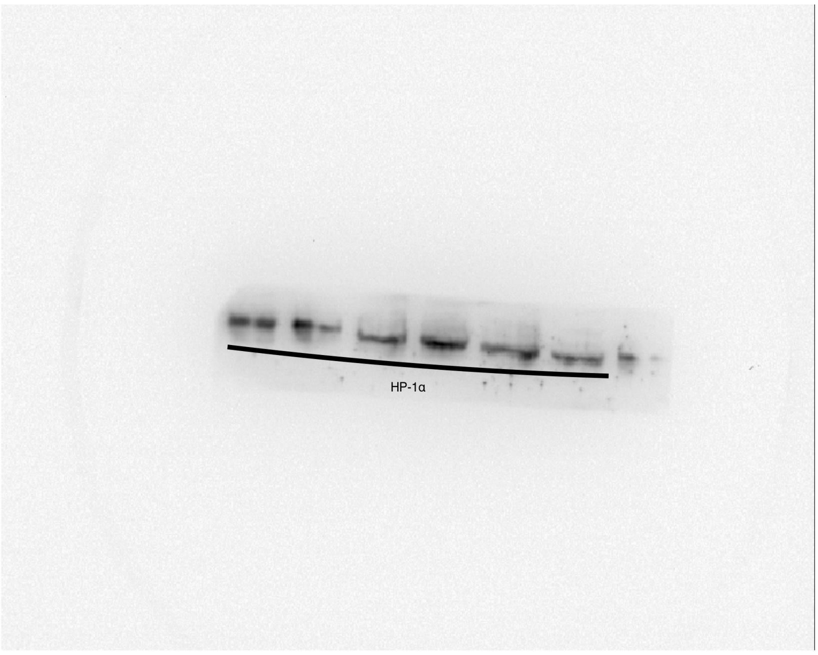

b

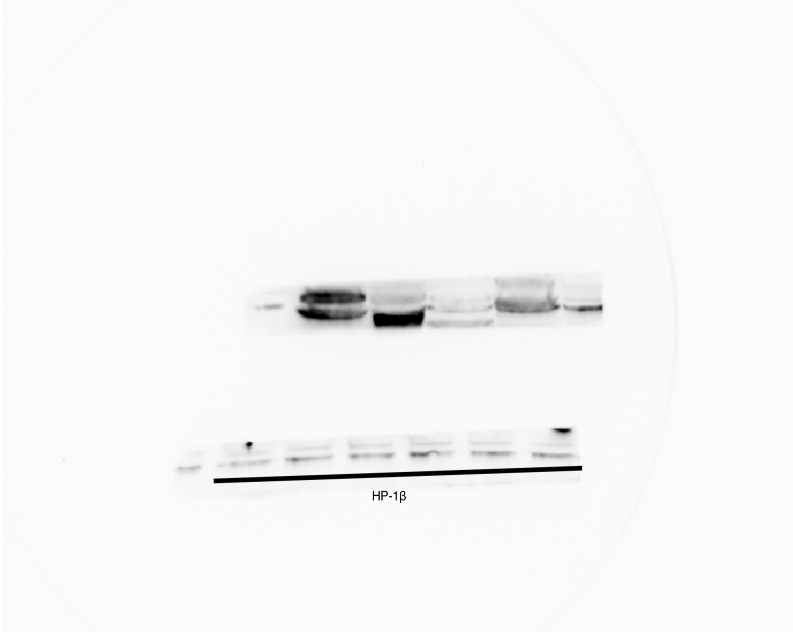

Supplementary Figure 5 Full-length blots/gels for Figure 4. (a) Corresponding to Figure 4a: HP-1α. (b) Corresponding to Figure 4a: HP-1β.

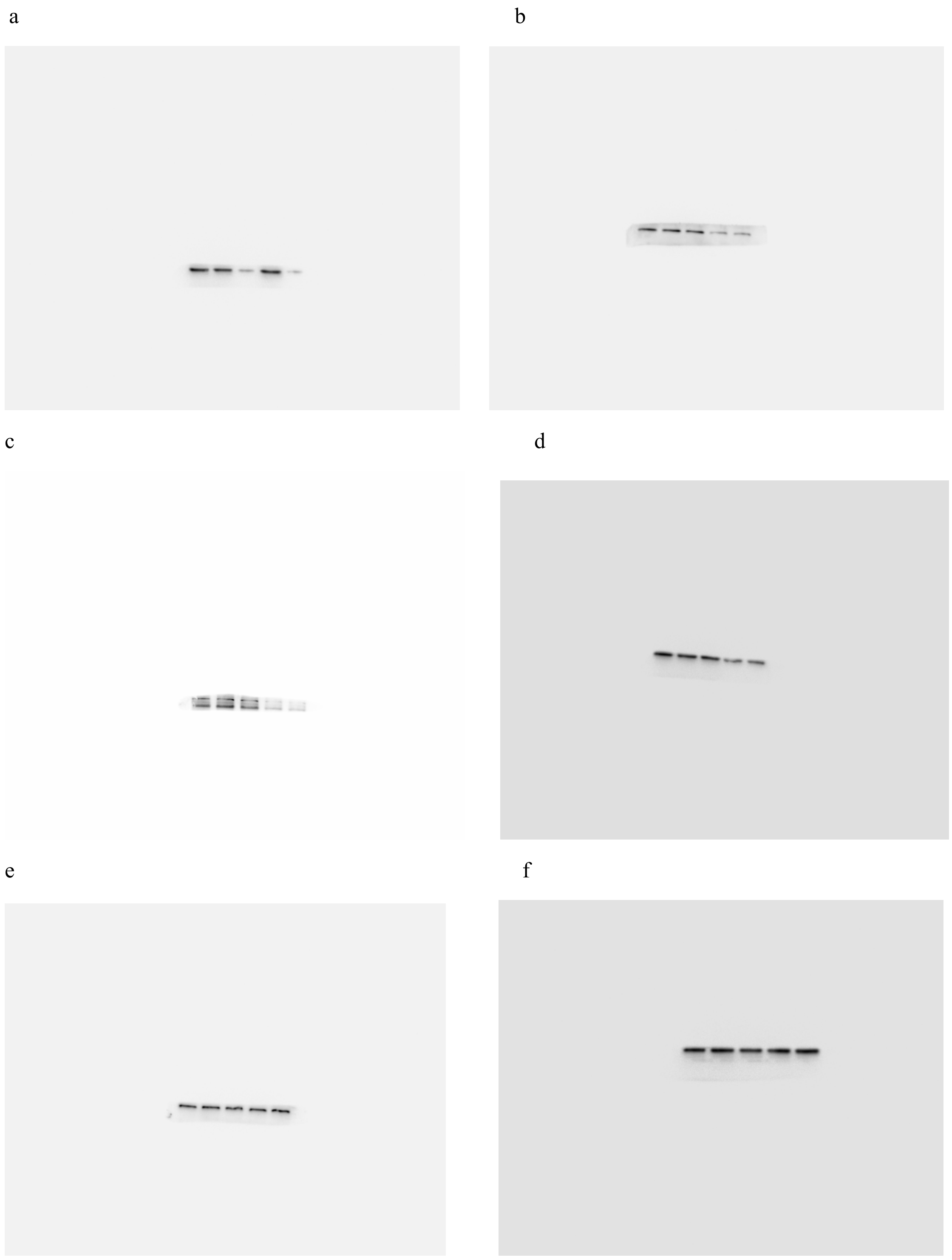

Supplementary Figure 6 Full-length blots/gels for Figure 5. (a) Corresponding to Figure 5a: Gcn5. (b) Corresponding to Figure 5a: HDAC-1. (c) Corresponding to Figure 5a: G9A. (d) Corresponding to Figure 5a: DNMT-1. (e) Corresponding to Figure 5a: HP-1α. (f) Corresponding to Figure 5a: HP-1β

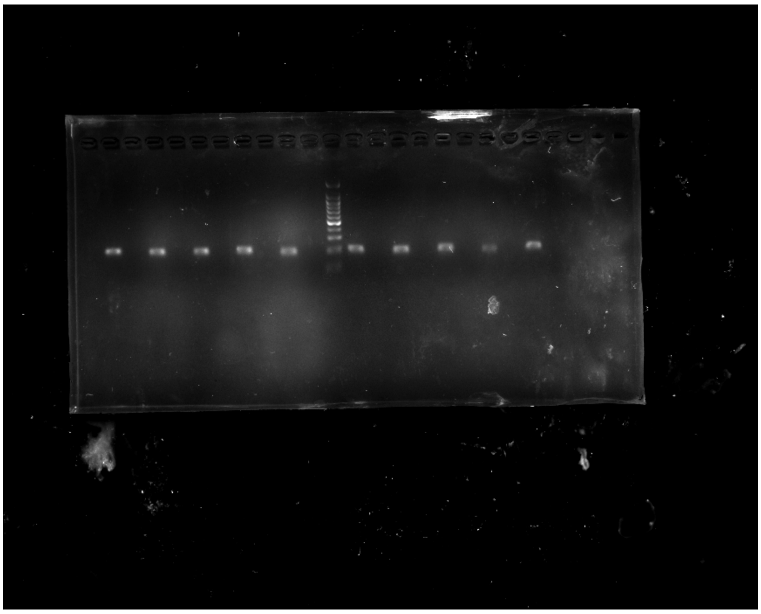

Supplementary Figure 7 Uncropped images of Figure 6 shown in the main manuscript. The right side of marker Corresponding to Figure 6b: GATA4. The left side of marker Corresponding to Figure 6b: Nkx2.5.

Supplementary Table 1. The antibodies used for Chip-qPCR & Western blotting.

Antibodies	Manufacturer	Cat. No.
Gcn5	Epigentek	A-4013
P300	Abcam	Ab14984
HDAC1	Abcam	Ab7028
HDAC2	Abcam	Ab51832
HDAC4	Abcam	Ab12171
G9A	Abcam	Ab185050
Suv39h1	Abcam	Ab12405
DNMT-1	Abcam	Ab13537
DNMT-3a	Abcam	Ab2850
DNMT-3b	Abcam	Ab2851
HP1-α	Abcam	Ab77256
HP1-β	Abcam	Ab10478
Connexin43	Abcam	Ab11370
cTnT	Abcam	Ab10214
β-actin	Sigma	A5441

Supplementary Table2. Actual p-values for all analysis in text.

	a		b		c		d		e	
	*	#	*	#	*	#	*	#	*	#
Figure2	-		Gcn5:0.025<br>P300:0.02	-	0.023	-	GATA4:0.021<br>Nkx2.5:0.019	-	GATA4(3w):0.012<br>GATA4(4w):0.014<br>Nkx2.5(3w):0.03<br>Nkx2.5(4w):0.016	-
Figure3	-		0.026	-	DNMT1:0.022<br>DNMT3a:0.041	-	GATA4-3w:0.011<br>GATA4-4w:0.015<br>Nkx2.5-3w:0.042<br>Nkx2.5-4w:0.015	-	0.021	-
Figure4	-		-		HP1α-3w:0.042<br>HP1α-4w:0.017<br>HP1β-3w:0.04<br>HP1β-4w:0.03	-	-		-	

Figure5	-		Gcn5(Control+MB-3):0.014	0.024	Gcn5(Control+MB-3):0.046	Gcn5:0.017	Gcn5(Control+MB-3):0.04	Gcn5:0.046	-
			Gcn5(LV-islet-1):0.027		Gcn5(LV-islet-1):0.04	HDAC1:0.036	Gcn5(LV-islet-1):0.031	HDAC1:0.017	
			HDAC1:0.03		HDAC1：0.049	G9A:0.029	HDAC1：0.016	G9A:0.029	
			G9A:0.024		G9A:0.025	DNMT1:0.041	G9A:0.027		
			DNMT1:0.018		DNMT1:0.018	HP1α:0.017			
					HP1α:0.028	HP1β:0.016			
					HP1β:0.021				
Figure6	GATA4(H3K9ac):0.019	GATA4(H3K9ac):0.028	-		-		-		-
	GATA4(H3K9me2:0.023	GATA4(H3K9me2:0.024							
	Nkx2.5(H3K9ac):0.029	Nkx2.5(H3K9ac):0.016							
	Nkx2.5(H3K9me2):0.046	Nkx2.5(H3K9me2):0.033							
Figure7	GATA4:0.029	GATA4:0.014	-		-		-		-
	Nkx2.5:0.014	Nkx2.5:0.035							
